# The Voice

**Published:** 1880-12

**Authors:** 


### THE VOICE.

Dr. Ward, of New York, says on this subject, of the many agents which have more or less influence on the voice, the four principal are climate, dress, diet, and exercise. Change of climate may cause some slight deleterious effect on the larynx, but this influence is greatly over-estimated. The present fashionable style of dress is decidedly unhealthy. The chest and abdomen are unnaturally confined, the lungs and other organs acting abnormally. All clothing should be loosely attached to the body, and the dress worn high. Avoid as much as possible appearing in full dress. The throat should not be wrapped in comforters, boas, etc. Chest protectors should not be worn, and the feet should be guarded against wet. The diet of the singer should be bland as well as nutritious. Of the different kinds of meat, venison, poultry, roast beef, and lamb are the easiest to digest, and due proportion of fat should be taken as a heat-supplying principle to the body. Cooked vegetables, unless too highly seasoned, are easily digested. Salads, cut cabbage, cucumbers, etc., should be avoided. Pastry should be invariably discarded. Dinner at noon, followed by a light tea at nightfall, is a rule, which, if rigidly adhered to, will be a safeguard against all ordinary attacks of indigestion. In order that the act of singing be properly performed, it is absolutely necessary that the stomach be nearly empty. Alcoholic beverages should not on any consideration be indulged in by vocal artists.

For the full development and preservation of the vocal cords several rules must be observed. The exercises must be regularly and systematically practiced; they must always be within the register; they should never be pushed to the point of fatigue; they should never be made use of when the vocal organs are attacked with cold, no matter how slight. Always practice standing upright, so as to allow of full play of the lungs and accessory vocal organs. Bodily exercise is especially beneficial to the singer. In short, learning to sing is learning to be healthy.—*The Monthly Magazine.*

